# CD8 T Cells and *Toxoplasma gondii*: A New Paradigm

**DOI:** 10.1155/2011/243796

**Published:** 2011-05-18

**Authors:** Jason P. Gigley, Rajarshi Bhadra, Imtiaz A. Khan

**Affiliations:** Department of Microbiology, Immunology and Tropical Medicine, George Washington University, Washington, DC 20037, USA

## Abstract

CD8 T cells are essential for control of *Toxoplasma gondii* infection. Once activated they undergo differentiation into short-lived effector and memory precursor effector cells. As effector cells, CD8 T cells exert immune pressure on the parasite via production of inflammatory cytokines and through their cytolytic activity. Once immune control has been established, the parasite encysts and develops into chronic infection regulated by the memory CD8 T-cell population. Several signals are needed for this process to be initiated and for development of fully differentiated memory CD8 T cells. With newly developed tools including CD8 T-cell tetramers and TCR transgenic mice, dissecting the biology behind *T. gondii*-specific CD8 T-cell responses can now be more effectively addressed. In this paper, we discuss what is known about the signals required for effective *T. gondii*-specific CD8 T-cell development, their differentiation, and effector function.

## 1. Introduction

Immune protection against many intracellular pathogens including viruses, bacteria, and protozoa is provided by robust CD8 T-cell responses. Naïve CD8 T cells are found in lymphoid tissues where, after infection, they encounter an antigen-presenting cell (APC) [1]. The APC presents pathogen-derived antigens and provides the appropriate costimulatory signals to the T cell to cause their activation [2, 3]. This activation leads to the proliferation, differentiation, and acquisition of effector functions of the antigen-specific CD8 T cell. Activated antigen-specific CD8 T-cell effector functions include secretion of cytokines IFN*γ* and TNF*α* and cytotoxicity, which promote further development of adaptive immunity and control pathogens. 

Original work by Frenkel revealed that antibodies, when transferred from infected to naïve hamsters, provided little protection against acute disease in the latter [4]. As such, he adoptively transferred intact or lysed spleen cells from infected animals to naïve animals then challenged them. He found that only intact cells were able to confer immunity to the recipient animals. Later, using the ts-4 model of infection developed by E. R. Pfefferkorn and L. C. Pfefferkorn [5], Suzuki and Remington [6] further dissected this immunity. Using an antibody depletion strategy with anti-CD4 and anti-CD8, they showed that both CD4 and CD8 T cells were important for control of infection with CD8 T cells playing the dominant role [6]. Further studies by this group using a similar strategy with anti-IFN*γ* and adoptive transfer of CD8 T cells identified that IFN*γ* was a major mediator of disease [7]. A short time later Khan and colleagues developed the first antigen-specific CD8 T cell clones capable of responding and killing *T. gondii* tachyzoites *in vitro* via their cytotoxic activity [8]. These studies suggested that two effector mechanisms may be at play in controlling *T. gondii* infection, IFN*γ* activation of macrophages, and cytotoxicity mediated by CD8 T cells. Subsequent studies reinforced the hypothesis that CD8 T cells were the dominant effector cell for control of * T. gondii* and were the source of IFN*γ* [9]. 

*Toxoplasma gondii* is an obligate intracellular parasite of the phylum apicomplexa which infects approximately 30–80% of humans world wide [10]. Primary infection during pregnancy causes severe birth defects and blindness in the developing fetus [11]. Infection of adults is largely asymptomatic during the acute stage of infection and once disseminated encysts in immune privileged sites including the brain where it persists for the life of the host. However, with loss of immune competence from HIV/AIDs or immunosuppressive therapies, the parasite can reactivate. Reactivation of the infection in the central nervous system results in the development of encephalitis termed toxoplasmic encephalitis (TE) and causes death of the host. 

*T. gondii* population structure can be generally divided into three clonal types based on virulence in mice [12, 13]. Recent studies now indicate that this population structure is much more diverse in places such as rural South America and in Africa with this diversity associated with increased risk of primary and reactive ocular toxoplasmosis [14–16]. Separately, *T. gondii* is also now being correlated with increased risk of personality changes and potentially enhanced development of dementia and Alzheimer's disease in the elderly [17–22]. Therefore, beyond development of TE in immunocompromised patients, immune-competent individuals may be at higher risk for complications associated with this infection.

Since these discoveries were made, the complexity behind activation, development, and differentiation of CD8 T cells into effector and memory cells and their functions in disease has increased greatly. In this paper, we present current knowledge of how signal one (antigen recognition), signal two (costimulation), and signal 3 (cytokines) affect activation of CD8 T-cell responses to *T. gondii * infection. We also review CD8 T-cell effector functions, the identification of different phenotypes of CD8 T cell, and how they may influence the pathogenesis of this disease.

## 2. Generation of CD8 T-Cell Responses

### 2.1. Signal One: Antigen Presentation and Recognition

One important cell-cell interaction between the APC and the CD8 T cell needed for activation is recognition of antigen presented on MHC Class I. Early studies using inbred and outbred mouse strains showed that there were different susceptibilities to *T. gondii* infection suggesting that beyond differences in inflammatory responses, a genetic component could influence the control of the parasite [23–25]. Susceptibility was shown to be controlled by MHC Class I haplotype by using mice with mutated and knockout mice for the L^d^ allele [26–28]. Mice that possess this allele are protected (Balb/c), while mice that are H-2^b^ (C57BL/6) are susceptible to infection. This allele specificity for control of *T. gondii* is also found within humans as shown by using mice expressing human MHC Class I transgenes [29]. Therefore, MHC Class I haplotype is important for the generation of optimal antigen-specific CD8 T-cell responses to *T. gondii* infection.

Antigen specificity is vital for the study of CD8 T-cell responses regardless of infection. For this reason, transgenic parasites expressing the model antigen ovalbumin have been developed [30]. While ova-transgenic parasites permit tracking of ovalbumin-specific CD8 T cells, recent studies in other models have suggested that pMHC-TCR avidity and affinity determine T-cell fate and functionality [31, 32]. This bears the implication that examining CD8 responses against ova, an exogenous protein may be useful for dissecting general CD8 biology; however, the insights gained from such a study may not necessarily be entirely applicable to the dynamics and quality of CD8 T-cell response specific for epitopes native to *T. gondii*. Recently, several *T. gondii*-specific antigens have been identified to be the source of peptides controlling dominant CD8 T-cell responses during infection. These include peptides derived from parasite proteins GRA6 (HF10), Tgd057, GRA4, and ROP7 [33–35]. Also, using epigenetic reprogramming by somatic cell nuclear transfer (SCNT) with CD8 T cells from *T. gondii*-infected mice, a CD8 T-cell TCR transgenic mouse has been developed for a parasite dominant antigen [36]. Despite the development of these tools, major questions remain including how effective these defined dominant CD8 T-cell responses are for control of infection as individuals and are CD8 T cells specific for multiple targets needed for immunity to this infection. For example, although HF10 peptide-BMDC immunization promoted survival against challenge infection, parasite burden was not measured [33]. Therefore, did this immunization reduce the overall parasite burden and development of chronic infection? GRA4- and ROP7-derived peptides also identify and stimulate significant antigen-specific CD8 T-cell responses after infection; however, whether these CD8 T-cell clones are effective at protection has not been tested [34]. Similarly, Tgd057 and the *T. gondii* TCR transgenic CD8 T cells have not been tested for direct control of the parasite *in vivo* despite providing increased survival after infection [35, 36]. The TCR transgenic CD8 T cells only prevent around 50–60% survival against challenge when adoptively transferred into naïve mice. Tgd057 CD8 T cells comprise only 2-3% of brain CD8 T cells and since access to the brain by CD8 T cells is restricted to antigen-specific cells only [37], this suggests that multiple epitopes are likely required for control of chronic *T. gondii * infection. Regardless, tetramer and transgenic TCR-based CD8 T-cell tracking will permit Toxoplasma immunologists to address complex questions regarding parasite-specific CD8 biology that could not be addressed as thoroughly before. The development of these tools will be essential for the future study of CD8 T-cell responses to *T. gondii* infection.

### 2.2. Signal Two: Costimulation

A second cell-cell interaction important for proper CD8 T-cell activation is mediated via costimulatory/coinhibitory molecules. In addition to recognition of antigen presented by the MHC class I molecules, CD8 and CD4 T cells require costimulation by the APC to become fully activated [1, 2]. Costimulation occurs mainly via interaction of two families of proteins, the immunoglobulin (Ig) superfamily members B7/CD28 and the TNFR/TNF superfamily of proteins [3]. Regulation of costimulation and thus the levels of CD8 T-cell activation can also occur via these families of proteins, more specifically by CD28 homolog cytotoxic T-lymphocytes-associated antigen 4 (CTLA4), inducible T-cell costimulator (ICOS), and programmed cell death 1 (PD1) [3, 38, 39]. Early investigations of how costimulation affects T-cell activation in response to *T. gondii* showed that CD86 played a more dominant role in this process [40]. Using human PBMCs, use of anti-CD86 reduced the level of T-cell activation in response to *T. gondii* stimulation whereas anit-CD80 had less effect. Using this same strategy, CD40 and CD40L were shown to play an important role in the activation of human T cells in response to *T. gondii *[41]. Interestingly, subsequent studies in mice revealed that CD28, a receptor for CD80 and CD86, was not required to generate T-cell responses yet contributed to the immunopathology in the brain [42]. Although there is no reduction in activation of T cells, CD28-deficient mice had a reduced ability to recall to subsequent challenge. In the following studies, CD86/CD28 and CD40/CD40L were found to control the acute inflammation and pathology in IL-10-deficient mice [43] suggesting that they may play a role in normal T-cell responses to *T. gondii* infection. Given that CD28-deficient mice appeared to be resistant to *T. gondii* infection, CD28-independent responses were investigated for their role in T-cell activation to parasite infection. These studies focused on the inducible costimulator protein (ICOS) as it is homologous to CD28. Unlike CD28, however, ICOS is an inducible costimulatory molecule for T-cell activation [44]. Despite CD28-deficient animals being resistant to *T. gondii* infection, blockade of ICOS *in vivo* made these mice more susceptible to infection by reducing the proliferation and level of IFN*γ* production by T cells [45]. Despite a lack of strong support for CD28 in the activation of T cells to *T. gondii* infection, recent studies using protein kinase c-theta- (PKC-*θ*-) deficient mice have shown that this molecule is essential for both CD4 and CD8 T-cell activation against this parasite [46]. PKC-*θ* is recruited into microclusters TCR/CD28 by CD28 allowing for the initial signaling needed for T-cell activation [47]. CD28 then retains PKC-*θ* in a subregion causing sustained signaling. Taken together whether or not CD28 is required for activation of CD8 T cells in response to *T. gondii* is still unclear; however, based on the PKC-*θ*-deficient mouse results, further exploration is needed to fully understand these signaling molecules in development of CD8 T-cell immunity to this parasite. Overall, little is known about costimulation and CD8 T-cell activation in response to* T. gondii* infection. As the complexities behind the development of quality of CD8 T cells grow, understanding the mechanisms behind costimulation and coinhibition is an important area of investigation. 

### 2.3. Signal Three: Cytokines

Several stimuli are required for the activation of CD8 T cells to proliferate and differentiate. Apart from antigen recognition and costimulation, signal 3, the cytokine milieu, plays a critical role in this process. Signal 3 is produced by many different cell types, and there are several inflammatory cell types that infiltrate into the site of infection and exert their function during *T. gondii* infection [48]. These are neutrophils (PMNs), macrophages, and dendritic cells. Each of these cell types contributes to the immune response against infection by providing cytokines and chemokines, toxoplasma-static and -cidal mechanisms of control, and in the case of macrophages and DCs, antigen presentation [49–57]. A key cytokine produced by these cells during initial infection and that is required for initiation of CD8 T-cell responses against *T. gondii* infection is IL-12 [58–61]. IL-12 is a heterodimeric protein formed by two subunits, IL-12p40 and IL-12p35, and when assembled together form the biologically active form of IL-12 defined as IL-12p70 [62]. IL-12p70 can induce activation of NK and T cells to produce IFN*γ* via a JAK/STAT-dependent pathway [58, 59, 63, 64]. Virulence level of *T. gondii* can alter the amount of IL-12p70 heterodimer and IL-12p40 monomer produced by different innate effector cells [65]. This likely leads to differences in the quality of CD8 T-cell responses produced, as with highly virulent strains, CD8 responses are largely impaired and correlate with high level IL-12p40 production [66]. This hypothesis is supported by work in other infection models showing that IL-12 influences the differentiation of newly activated CD8 T cells leading to different levels of CD8 T-cell memory development [67, 68]. Recent investigations of the function of IL-12 in CD8 T-cell differentiation during *T. gondii* infection largely support that this cytokine is vital for this process [69]. 

Several cytokines, other than IL-12, are important for CD8 T-cell function in response to *T. gondii* infection including common-cytokine-receptor *γ*-chain cytokines including IL-2, IL-7, and IL-15. Whether or not IL-2 plays a role in CD8 T-cell activation in response to *T. gondii* remains unknown. Very little work has been done to investigate the function of IL-7 in the development of CD8 T-cell responses. IL-15, a member of the common-cytokine-receptor *γ*-chain family of cytokines, is important for the maintenance of long-lasting high avidity memory CD8 T-cell populations [70]. IL-15 has been shown to enhance CD8 T-cell responses, and blockade of this cytokine with a soluble receptor abrogates immunity [71–73]. Surprisingly, IL-15 knockout mice survive as well as wild type mice when infected with *T. gondii* [74, 75]. However, in the absence of both IL-7 and IL-15, CD8 T-cell responses are severely impaired and mice do not survive [76]. In addition to cytokines, interactions with APCs are also very important in the activation of CD8 T cells. 

### 2.4. Effector Function and Differentiation of CD8 T Cells

The downstream consequence of properly coordinated Th1 activation signals is the development of robust long-term CD8 T-cell memory. After activation of naïve CD8 T cells during an infection, they rapidly proliferate and differentiate into a heterogeneous population of effector cells composed of terminally differentiated effector cells termed short-lived effector cells (SLECs) and cells that are more long lived and are the precursors to memory cells termed memory precursor cells (MPECs) [77]. The SLECs typically provide the mechanism of control via their production of effector molecules including cytokines and cytolysis. After this initial expansion and differentiation, the pool of activated CD8 T cells undergoes a process termed contraction where the terminally differentiated SLECs apoptose leaving behind the smaller subset of MPECs [77]. These memory precursors further differentiate into bona fide memory cells that undergo homeostatic proliferation and continually renew themselves. Many factors including antigen exposure, costimulation, and the level of inflammation influence the path these cells take. The mechanisms governing these processes are a major focus in immunology and are only now beginning to be addressed in the *T. gondii* field. 

### 2.5. CD8 T-Cell Effector Function

The major mediator of resistance to *T. gondii* infection is IFN*γ* [7]. IFN*γ* can be produced by several cell types in response to parasite infection including NK, NKT, *γδ* T, CD4, and CD8 T cells [9, 58, 78–81]. CD8 T-cell production of this cytokine is essential for their ability to control the parasite [9]. IFN*γ* produced by CD8 T cells acts on surrounding cells, including macrophages, which in turn inhibits the proliferation of the parasite via induction of inducible nitric oxide synthase (iNOS) and indoleamine 2,3-dioxygenase (IDO) [54, 82–90]. These two enzymes control the parasite via toxicity of nitric oxide and starvation of the parasite by removal of the amino acids L-arginine and tryptophan for which *T. gondii* has a natural auxotrophy [86, 91]. CD8 T cells produce IFN*γ* in response to IL-12-induced T-bet expression [59, 61, 67, 69]. Kinetically, CD8 T-cell production of IFN*γ* begins 2-3 days after parasite infection with eventual contraction out to 21 days after infection followed by low numbers being maintained [66, 69, 92]. CD8 T-cell-derived IFN*γ* then is likely to have two roles in controlling the parasite, initially during acute infection to reduce parasite numbers and then during chronic infection to exert immune pressure sufficient to maintain the parasite encysted as a bradyzoite. This has been previously established where anti-IFN*γ*-treated WT or IFN*γ* KO (GKO) mice result in death 8 days later due to uncontrolled acute infection, and anti-IFN*γ* treatment of chronically infected mice results in reactivation of encysted parasites [7, 48, 93, 94]. Interestingly, we and others have found that only exceedingly high levels of anti-IFN*γ* and upwards of 6 mg/mouse are required to cause parasite reactivation [93]. Even in cases where this reactivation is initiated, we have noticed that mice recover from this treatment (unpublished observations). Therefore, additional CD8 T-cell effector mechanisms are likely required for the control of the parasite in both acute and chronic infections. Another interesting question remaining to be answered is whether or not IFN*γ* is required for the development of CD8 T-cell responses during *T. gondii* infection. IFN*γ* is known to signal through STAT1 resulting in T-bet-dependent upregulation of the IL-12R*β*1 [95]. Therefore, IFN*γ*, in an autocrine fashion, may also be important for CD8 T-cell differentiation. Investigation of this possibility could be done with the current parasite tools and IFN*γ*R1 KO mice. 

CD8 T cells can also provide effector function via the production of other cytokines including TNF*α* and through their cytolytic abilities [96]. The role of TNF*α* in the control of *T. gondii* infection was shown early on to be synergistic with IFN*γ* [97]. For macrophages to control the growth and replication of *T. gondii* parasites, they first needed priming by IFN*γ* then required TNF*α*. A second study investigated whether the lack of TNF*α* had an effect on the survival of either toxoplasmic encephalitis- (TE-) resistant or susceptible mice [98]. This study revealed that when infected per orally, C57BL/6 mice died when treated with anti-TNF*α*. However, in a TE-resistant strain of mice, anti-TNF*α* treatment had no effect. An additional study investigating the cause of TE in mice revealed that TNF*α* transcripts were elevated after infection in the brain [99]. As the mice succumbed to TE, the level of specific mRNA transcripts, including TNF*α*, was reduced. Therefore, they treated mice with anti-TNF*α* after chronic infection was established (4 weeks p.i.) and measured their survival. Interestingly, they found that treatment with anti-TNF*α* caused reactivation of *T. gondii* as quickly as anti-IFN*γ* treatment, suggesting that this cytokine played an important role in the control of *T. gondii* as the latter. Subsequent studies showed that the TNF*α*-dependent antitoxoplasma effect was derived from both hematopoietic and nonhematopoietic sources [100–103]. Combined together, these studies reveal an important function of TNF*α* in the control of *T. gondii* parasites and development of TE in mice. However, whether CD8 T cells are directly producing this TNF*α* in response to infection has not been tested. Defining which CD8 T-cell populations are important for delivering this effector function will be important to address in future studies. 

CD8 T cells have been shown to directly kill extracellular tachyzoites of *T. gondii* in culture [8]. CTL clones and CTLs from mice vaccinated with the temperature-sensitive mutant (ts-4) were also able to kill parasitized and parasite antigen-pulsed target cells *in vitro* [104, 105]. Cytotoxicity is also exhibited by human CD8 T cells and can kill antigen-pulsed cells *in vitro* [106, 107]. Despite the findings from these papers, the role of the cytotoxic CD8 T cell in the control of *T. gondii* infection is still unclear. Although immunization of perforin knockout mice (PKO) resulted in defective cytolysis of antigen-pulsed target cells, when challenged with a type I strain of parasite, these mice survived challenge infection [108]. Subsequent studies have supported these findings and have shown that IFN*γ* not perforin or CTL activity is required for immune protection against *T, gondii* infection [109]. Recent studies using an antigen-specific OVA transgenic parasite system and perforin knockout mice suggest that the more prominent mechanism of CTL activity was perforin dependent* in vivo* and that Fas-FasL played a subordinate role [92]. Although this study did not investigate whether perforin and CTL activity was a major mechanism of control, a subsequent study reports that adoptive transfer of immune CD8 T cells into chronically infected SCID mice (treated with sulfadiazine to establish chronic infection) controlled and removed encysted parasites in the brain via a perforin-dependent mechanism [110]. Taken together, research of effector functions of CD8 T cells important for the control of *T. gondii* infection has clearly established that IFN*γ* is required. Whether or not TNF*α* or CTL activity is needed for initial control, for development of CD8 memory, or required for long-term control of chronic infection needs more investigation. With new tools to track antigen-specific CD8 T cells including TCR transgenic mice and transgenic parasites expressing OVA protein, investigations of what roles different effector mechanisms play in control of *T. gondii* are now feasible. 

## 3. Roles of SLEC and MPEC in *T. gondii* Infection

SLEC and MPEC can be generally distinguished by their phenotype based on their surface expression of Killer cell lectin-like receptor G1 (KLRG1) and IL-7R*α* (IL-7R) [68]. SLECs are defined as being KLRG1^hi^ and IL-7R^Io^ whereas MPECs are KLRG1^Io^ IL-7R^hi^. Despite their difference in phenotype, these CD8 T-cell populations produce similar levels of effector functions including IFN*γ*, cytotoxic activity, and proliferation [68]. Interestingly, the level of IL-12 had a profound effect on their development. Previous studies had shown that in the absence of IL-12, CD8 T cells differentiated into more memory cells after infection with *Listeria monocytogenes *[67]. Similar to this study, Joshi et al. [68] showed that the level of IL-12 correlated with the amount of the Th1 transcription factor T-bet and that higher T bet resulted in greater SLEC and less MPEC differentiation. The converse was also true in that lower levels of IL-12 resulted in higher MPEC and lower SLEC differentiation. Original studies with *T. gondii* broadly defined effector populations by their ability to proliferate, be cytotoxic, and produce IFN*γ* and memory populations on their ability to recall to challenge or antigen. Recent work on CD8 T-cell effector differentiation in response to *T. gondii* has revealed that IL-12 is required for SLEC generation [69]. This study agrees with previously published reports using other models of infection that in the absence of IL-12 using IL-12p35KO mice, MPECs were present at a higher frequency than SLECs. The *T. gondii* study, however, showed that effector generation was solely dependent upon CD8 T-cell intrinsic IL-12 signaling and unlike viral and bacterial models was independent of CD4 T-cell help [69]. This is also in contrast to a previous report using OVA-expressing transgenic parasites where CD4 T-cell help was reported to be required for effector CD8 T-cell generation [92]. Regardless, the generation of SLEC and MPEC in response to *T. gondii* infection appears to be solely dependent upon intrinsic IL-12 signaling. As parasite virulence can alter the levels and types of IL-12 produced during infection, one mechanism of escape may be via the overproduction of IL-12, thereby inhibiting the development of MPEC important for the control of chronic infection. Recent reports may support this hypothesis where treatment of mice with rIL-12p70 partially rescues antigen-specific CD8 T-cell responses in mice infected with highly virulent parasite strain [111]. 

SLECs required IL-15 for survival in response to LCMV infection most likely due to their inability to receive prosurvival signals from IL-7 because of their low expression of IL-7R [68]. IL-15 knockout mice have been shown to be able to survive *T. gondii* infection as well as WT mice with near normal activation of their CD8 T cells [66, 74, 76]. Interestingly, CD4 T-cell priming is defective in these mice [75]. During *T. gondii* infection, CD8 T-cell activation does not require CD4 T-cell help; however, CD4 T cells are required to maintain memory CD8 T-cell responses [112]. It is surprising then that IL-15 KO mice do not succumb more rapidly to long-term infection with *T. gondii*, given that CD4 T-cell help has been shown to be required for long-term CD8 T-cell memory responses to LCMV and *Listeria monocytogenes* [113, 114]. One possible explanation for these results could be a result of the mouse strain used for these studies. C57BL/6 mice are most often used to study basic immunology in response to *T. gondii*. They are inherently unable to fully control the parasite due to their MHC Class I allele [27, 28]. Due to this defect, they may be less efficient at developing memory CD8 T-cell responses while having continuous generation of SLEC responses. A lack of memory CD8 T-cell differentiation could explain then the susceptibility of these mice, a result of the eventual development of exhaustion in the responding CD8 T-cell population [115]. Major questions still need to be addressed about which CD8 population (SLEC or MPEC) is important for ultimate control of the parasite and how this relates to evasion of the parasite from immune surveillance. With the recent identification of dominant antigens and tools for detecting antigen-specific CD8 T cells, investigation of how CD8 T cells are activated, how they undergo their initial differentiation, and which population is important in controlling infection will be much easier [33–36]. 

## 4. Concluding Remarks

There is little doubt that CD8 T cells are central for the control of* T. gondii* infection and, in particular, their production of IFN*γ*. Many factors play a role in the development of this response; however, the body of knowledge behind CD8 T-cell responses to *T. gondii* infection to date remains very superficial especially in regard to the molecular mechanisms behind their initial activation, differentiation, and memory development. Given the breadth of information on the complexities underlying the biology of CD8 T cells obtained using other intracellular pathogens including bacterial and viral models of infection, major gaps exist in the current understanding of their role in *T. gondii* infection. Many questions still exist in areas such as, but not restricted to, (1) how the array of recognizable antigens that stimulate CD8 T cells affects this response, (2) how signals 1, 2, and 3 alter the differentiation of the different CD8 T-cell subpopulations, (3) molecular pathways involved in the programming of these cells, (4) the required effector functions and when and where they are important, (5) the role of accessory cells including CD4 T-cell help, and (6) the identification of mechanisms behind the development and maintenance of effector memory and central memory CD8 T-cells populations. Additional areas are in need of investigation center on how parasite biology can affect this response. Protozoan biology is exceedingly complex and can profoundly influence the response of the host and represents a major barrier in the development of immunity. How these parasite mechanisms alter host immunity and how this affects development of CD8 T-cell effector function and memory is unknown. The evidence for this parasite-driven immune evasion is present in that despite the development of powerful strategies to create a vaccine against this disease, the parasite continually escapes and establishes a chronic infection. Therefore, further investigation is needed to dissect both host and parasite biology to aid in the development of CD8 T-cell-based immunotherapies against this disease. 
